# [Retracted] MicroRNA-206 exerts anti-oncogenic functions in esophageal squamous cell carcinoma by suppressing the c-Met/AKT/mTOR pathway

**DOI:** 10.3892/mmr.2025.13762

**Published:** 2025-11-25

**Authors:** Jin Zhang, Xianen Fa, Qingyong Zhang

Mol Med Rep 19: 1491–1500, 2019; DOI: 10.3892/mmr.2018.9775

Following the publication of the above paper, it was drawn to the Editor's attention by a concerned reader that certain of the flow cytometric data shown in Figs. 2D and 4D, and western blot data shown in Fig. 5A, were strikingly similar to data that had already been published previously in articles submitted to different journals that were written by different authors at different research institutes, a few of which have been retracted. Owing to the fact that the contentious data in the above article had already been published prior to its submission to *Molecular Medicine Reports*, the Editor has decided that this paper should be retracted from the Journal. The authors were asked for an explanation to account for these concerns, but the Editorial Office did not receive a reply. The Editor apologizes to the readership for any inconvenience caused.

